# Changes of sexual risk behaviors and sexual connections among HIV-positive men who have sex with men along their HIV care continuum

**DOI:** 10.1371/journal.pone.0209008

**Published:** 2018-12-12

**Authors:** Chin Man Poon, Ngai Sze Wong, Tsz Ho Kwan, Horas Tze Hoo Wong, Kenny Chi Wai Chan, Shui Shan Lee

**Affiliations:** 1 Stanley Ho Centre for Emerging Infectious Diseases, The Chinese University of Hong Kong, Hong Kong; 2 The Jockey Club School of Public Health and Primary Care, The Chinese University of Hong Kong, Hong Kong; 3 Centre for Social Research in Health, University of New South Wales, Sydney, Australia; 4 Integrated Treatment Centre, Centre for Health Protection, Department of Health, Hong Kong; Lluita contra la SIDA Foundation - Germans Trias i Pujol University Hospital - Autònoma de Barcelona University, SPAIN

## Abstract

**Background:**

While HIV incidence among men who have sex with men (MSM) is increasing in Hong Kong, unprotected sex apparently remains prevalent among those infected but virally non-suppressed. Little is known about how sexual behaviours and sexual connections may change among MSM along their HIV care continuum.

**Methods:**

In this retrospective cross-sectional study, HIV-positive MSM attending the largest HIV specialist clinic in Hong Kong between October and December 2014 were invited to complete a self-administrated structured questionnaire. Their behavioural profile and partner sourcing patterns during the one-year period respectively (a) before HIV diagnosis, (b) after HIV diagnosis, (c) after initiation of antiretroviral treatment and (d) preceding the survey were examined.

**Results:**

Of 345 recruited MSM, 304 (88.1%) had treatment initiated and 272 (78.8%) had viral load suppressed. In the first year after HIV diagnosis, the proportion reporting inconsistent condom use dropped from 47.0% to 17.5% (p<0.05) and from 49.6% to 17.8% (p<0.01) for anal sex with main and casual partners respectively. Except for mobile applications, usage of most sex-networking venues decreased significantly after diagnosis. Inconsistent condom usage rate remained at around 20% after treatment initiation and viral load suppression, but the frequency of use of sex-networking venues further varied among virally suppressed MSM.

**Conclusions:**

Most HIV-positive MSM had persistently low level of sexual risk behaviours along their care continuum and achieved viral load suppression, conferring a general reduction of secondary transmission risk in Hong Kong. To increase the effectiveness of Treatment as Prevention strategy, uptake of HIV testing for undiagnosed HIV-positive MSM shall be emphasised.

## Introduction

Antiretroviral therapy (ART) can suppress the viral load of people living with HIV to an undetectable level, a phenomenon that decreases potential risk of secondary transmission [1]. This has become the rationale for a new public health strategy, named Treatment as Prevention (TasP). Its effectiveness in the community was suggested, following the demonstration of an association between antiretroviral programme and reduction of HIV incidence in some populations [2–5]. Despite the widespread use of ART for people living with HIV, an increase in HIV incidence among men who have sex with men (MSM) was observed in the past decade in western as well as Asian jurisdictions, the latter including Hong Kong [6–8]. The higher per act transmission risk of unprotected receptive anal intercourse, suboptimal coverage of testing and treatment for the hidden MSM populations, and the phenomenon of risk clustering within networks underlined MSM’s exposure risk to HIV, imposing a disproportionate virus burden in the community [9–10]. Effectiveness of the TasP strategy would therefore hinge on the inter-play among high risk behaviours, viral load suppression and changes in the sexual connections among MSM before and after HIV diagnosis.

Over the years, new infections through male-to-male sexual contact have continued to be reported, implying that unprotected sex remains prevalent among some HIV-positive MSM, especially those with non-suppressed viral load. A thorough understanding of the pattern of risk behaviours among HIV-positive MSM, both before and after their diagnosis, would be crucial to assess the potential risk of HIV spread in the community. Many studies have reported sharp declines in the number of sex partners after HIV diagnosis, alongside inconsistent condom use for anal intercourse and other sexual risk behaviours [11–16]. Transmission risk to HIV-negative persons should theoretically become lower with these behavioural changes adopted by newly diagnosed MSM. However, both the prevalence and persistence of reduced level of risk behaviours among HIV-positive MSM remains unclear in light of ongoing HIV transmission in some countries and globally. To date, very few studies had investigated the temporal changes of high-risk behaviours among HIV-positive MSM over an extended period [17–20]. Particularly, little is known if any of the high-risk behaviours persist at specific stages along the HIV care continuum, an internationally-recognised framework of the spectrum of engagement in HIV care, spanning from HIV diagnosis, linkage to care, initiation of ART to viral load suppression. [21–22]. The idea that risk behaviours might change at specific points across the HIV care continuum is in parallel with some recent studies assessing behaviour change following HIV seroconversion [23–24]. Moreover, it is anticipated that even the same level of risk behaviour might carry dissimilar implications on the potential risk of future HIV transmission for those in different stages of the cascade. Notably, the evidence that HIV-positive people who are on ART and virally suppressed cannot sexually transmit the virus to the others has led to changes in HIV prevention measures, signified by the launch of Undetectable = Untransmittable (U = U) campaign [25]. Still, virally suppressed HIV-positive people remain susceptible to other sexually transmitted infections (STI). Profiling how risk behaviours change across the HIV care continuum would be important for understanding both HIV and STI epidemiology.

Apart from risk behaviours, sex partnership and networking patterns of MSM before and after HIV diagnosis form another important dimension of HIV epidemiology. In Hong Kong where male-to-male HIV transmission has accounted for a significant proportion of local infections, its prevalence among MSM remained at a relatively low level at 4% between 2006 and 2011 [6, 26]. However, the latest community-based survey, conducted in 2014, showed an increased HIV prevalence at 5.85% [6]. This figure may escalate further if HIV-positive MSM are unaware of the HIV status of their sex partners and engage in social networks with common practice of high-risk behaviours. Research examining the influence of sero-sorting and network effects on HIV transmission dynamics has suggested the potential of sexual harm reduction following modification of sex-networking behaviours after HIV diagnosis [10,13,17,27]. In the current era of universal antiretroviral treatment, it is not known if HIV-positive MSM in some stages of the HIV care continuum were members of high-risk networks. An understanding of the network configuration of HIV-positive MSM would be essential to elucidate how HIV spreads in the population.

Focusing on Hong Kong, a retrospective cross-sectional study was conceptualised to assess the changes of usage of different social venues for sex-networking and the practice of sexual risk behaviours after infection with HIV. The study would particularly address how progressing along the HIV care continuum, rather than just time since HIV diagnosis, may be associated with sexual risk behaviours and sexual connections. It was hypothesised that the sexual partnership, network configuration and behaviour practice would be evolving over time.

## Materials and methods

Subject recruitment for this study took place at Integrated Treatment Centre, the largest HIV specialist clinic that took care of about 60% of the HIV clinical caseload in Hong Kong. Eligible subjects were male HIV patients who (a) were aged 18 or above, (b) had contracted HIV through male-to-male sexual contact, (c) could understand Chinese or English, and (d) normally resided in Hong Kong. HIV diagnosis was made by a combination of ELISA screening test and a western blot confirmatory test. During a two-month study period, research staff assessed the eligibility of each patient attending the clinic and invited potential participants to complete a self-administered questionnaire, embedded in a tablet computer. Informed written consent was obtained from the participants before they accessed the questionnaire. Ethical approval had been obtained from Joint Chinese University of Hong Kong–New Territories East Cluster Clinical Research Ethics Committee and the Ethics Committee of Department of Health. The ethics committees approved this consent procedure.

The questionnaire was constructed in both Chinese and English language and divided into five parts, the first of which covered demographics of the respondents. The latter four parts of the questionnaire comprised items about respondents’ sexual partnership characteristics, behavioural profile and partner sourcing patterns during the following four periods, namely the one-year period respectively (a) before HIV diagnosis, (b) after HIV diagnosis, (c) after initiation of ART, and (d) preceding the survey. In this study, partner sourcing referred to the frequency of using any one or more of eight identified types of sex-networking venues or channels for seeking male sex partners: (a) public toilets, (b) bars, (c) saunas, (d) parties, (e) swimming pools or beaches, (f) gymnasium, (g) mobile phone applications, and (h) websites, forum or chatroom. Respondents were also asked to report their frequency of anal intercourse and respective use of condom with different types of partners. Taking reference from our previous work, inquiries were made in the questionnaire in regards to three main types of sex partners, which were lovers, regular sex partners and non-regular sex partners [28]. Lovers were sex partners, whom the respondents were emotionally attached to. Regular sex partners referred to those with sexual relationship alone over extended periods, whereas non-regular sex partners were typically one-night-stand partners without extended relationship. For statistical presentation, three types of sex partners were recategorised into two groups using a more common distinction, namely main and casual sex partners [29]. Lovers and regular sex partners were considered as main sex partners, with whom the respondents felt committed or had an extended relationship. Casual sex partners referred to non-regular sex partners, with whom there was no commitment or who were not well known.

In the questionnaire, condom use was reported as an ordinal-scaled frequency variable, categorised as (a) always, (b) more than half of those occasions, (c) less than half of those occasions, and (d) never. Condom use was dichotomised in the statistical analysis. Inconsistent condom use was defined when the respondents did not always use a condom in the corresponding period included in the questionnaire. Inconsistent condom usage rate, thus, referred to that of respondents who did not respond “always use a condom”. Three other sexual risk behaviours were assessed, including (i) presence of concurrent sexual partnership, (ii) alcohol use before anal sex with male and (iii) recreational drug use before anal sex with male. Concurrency of sexual partnership referred to the establishment of regular sexual relationship with more than one man simultaneously. Alcohol use was defined as the consumption of any alcoholic drinks before having anal sex with male. Recreational drug use was defined as the intake of psychoactive or non-psychoactive drugs for the purposes of facilitating and/or enhancing sexual experience. These drugs might include, but not limited to, amphetamine, cannabis, cocaine, crystal methamphetamine, ecstasy, gamma-hydroxybutyrate (GHB) and ketamine.

Descriptive statistics of respondents’ demographics and prevalent sexual behaviours following HIV infection were tabulated. Statistical comparisons of sexual behaviours among MSM were also made based on their stages along the HIV care continuum. These stages referred to (a) undiagnosed, (b) recently diagnosed, (c) diagnosed and ART initiated, and (d) diagnosed, treated and viral load suppressed (≤500 copies/mL). For those having ART initiated in the same year as that of HIV diagnosis, post-treatment behaviours were assumed to be the same as that for post-diagnosis behaviours. Dates of viral load suppression of the respondents were extracted from the clinical database in Integrated Treatment Centre. Changes in sexual behaviours between two consecutive stages were tested by a modified McNemar’s test proposed by Yang [30]. Yang’s test was designed for clustered matched-pair binary data, allowing us to compare the changes in sexual behaviours of MSM stratified by age of HIV diagnosis (<35 years old / ≥ 35 years old), socio-economic status (working / non-working population), year of HIV diagnosis (before 2011 / 2011 or thereafter) and interval between HIV diagnosis and ART initiation (less than 2 years / 2 years or more). Statistical differences were classified as significant when *P* value was less than 0.05. All statistical analyses were conducted in R 3.4.0.

## Results

### Characteristics of respondents

Between October and December 2014, a total of 387 HIV-positive patients were approached for participation in the study. Of these, 345 completed the questionnaires and were included in the analyses. Respondents’ demographics, status in the HIV care continuum and sexual behaviours in the year preceding the survey are shown in Table 1. The mean age of the respondents was 37.8 years (standard deviation = 9.95 years). The majority of them (305/345; 88.4%) were part of the working population and about two thirds of the respondents (227/345; 65.8%) had attained post-secondary education.

**Table 1:** Demographics of respondents and their prevalent sexual behaviours following HIV infection.

Characteristics	Number (%) of respondents (n = 345)	
**Demographics**		
***Age***		
< 30	86	(24.9)
30–39	111	(32.2)
> = 40	148	(42.9)
***Current socio-economic status***		
Working population	305	(88.4)
Non-working population	40	(11.6)
***Education level attained***		
Secondary or below	118	(34.2)
Post-secondary	227	(65.8)
**HIV care cascade**		
***Time of last HIV testing before diagnosis***		
Within a year	96	(27.8)
More than a year ago / Never tested	249	(72.2)
***Year of HIV diagnosis***		
Before 2011	154	(44.6)
In 2011 or thereafter	191	(55.4)
***Age at HIV diagnosis***		
< 35 years old	207	(60.0)
> = 35 years old	138	(40.0)
***Treatment status***		
ART initiated, and viral load suppressed	272	(78.8)
ART initiated, and viral load not yet suppressed	32	(9.3)
ART not yet initiated	41	(11.9)
**Sexual behaviours in the past year**		
***Having main sex partner(s)***	207	(60.0)
***Having casual sex partner(s)***	164	(47.5)
***Inconsistent condom use for anal sex with main sex partner(s)***	67	(19.4)
***Inconsistent condom use for anal sex with casual sex partner(s)***	65	(18.8)
***Preference for sex partners***		
HIV-negative sex partners	41	(11.9)
HIV-positive sex partners	64	(18.6)
Did not care the HIV status of sex partners	203	(58.8)
Did not have sex any more	37	(10.7)
***Venues for sex-networking***		
Public toilets	29	(8.4)
Bars	83	(24.1)
Saunas	147	(42.6)
Parties	47	(13.6)
Swimming pools / beaches	74	(21.4)
Gymnasium	80	(23.2)
Mobile phone applications	199	(57.7)
Social networking websites / forum / chatroom	133	(38.6)

A snapshot of the status of the HIV care continuum, from diagnostic testing to life-long ART, could be illustrated by the following key indicators, (a) the time of last HIV testing before diagnosis, (b) age and year of HIV diagnosis, and (c) status for treatment initiation and viral load suppression. In this study, three fifths of the respondents (207/345; 60.0%) were diagnosed with HIV infection at the age below 35. The coverage of ART was approaching 90% (304/345) among the respondents, and almost 90% of those on treatment had their viral load suppressed (272/304).

Some 60% of the respondents (207/345) had main sex partners in the past one year, while less than half of them (164/345; 47.5%) had casual sex partners. About one fifth of the HIV-positive MSM reported that they did not always use a condom for anal sex with main partners (67/345; 19.4%) and casual partners (65/345; 18.8%) respectively. A similar proportion of them (64/345; 18.6%) indicated their inclination for seeking an HIV-positive sex partner, though the majority (203/345; 58.8%) were not concerned about the HIV status of sex partners. Mobile phone applications had emerged as the most popular means for sex-networking among HIV-positive MSM (199/345; 57.7%). Another popular venue for sex-networking was sauna, where 42.6% (147/345) of HIV-positive MSM had visited in the past year.

A total of 73 respondents were virally non-suppressed at the time of the survey, either because ART had not been initiated yet or it was too soon to have the viral load suppressed with ART. Of these, a smaller proportion had main sex partners [n = 41 (56.2%) vs. n = 166 (61.0%); *P* = 0.17] or casual sex partners [n = 29 (39.7%) vs. n = 135 (49.6%); *P* = 0.53] in the previous year before the survey, as compared with virally suppressed respondents. Their proportion of having inconsistent condom use was also lower for anal sex with main sex partners [n = 8 (11.0%) vs. n = 59 (21.7%); *P* = 0.06] and casual sex partners [n = 9 (12.3%) vs. n = 56 (20.6%); *P* = 0.15] respectively.

### Changes of sexual behaviours along the HIV care continuum

**Fig 1:**
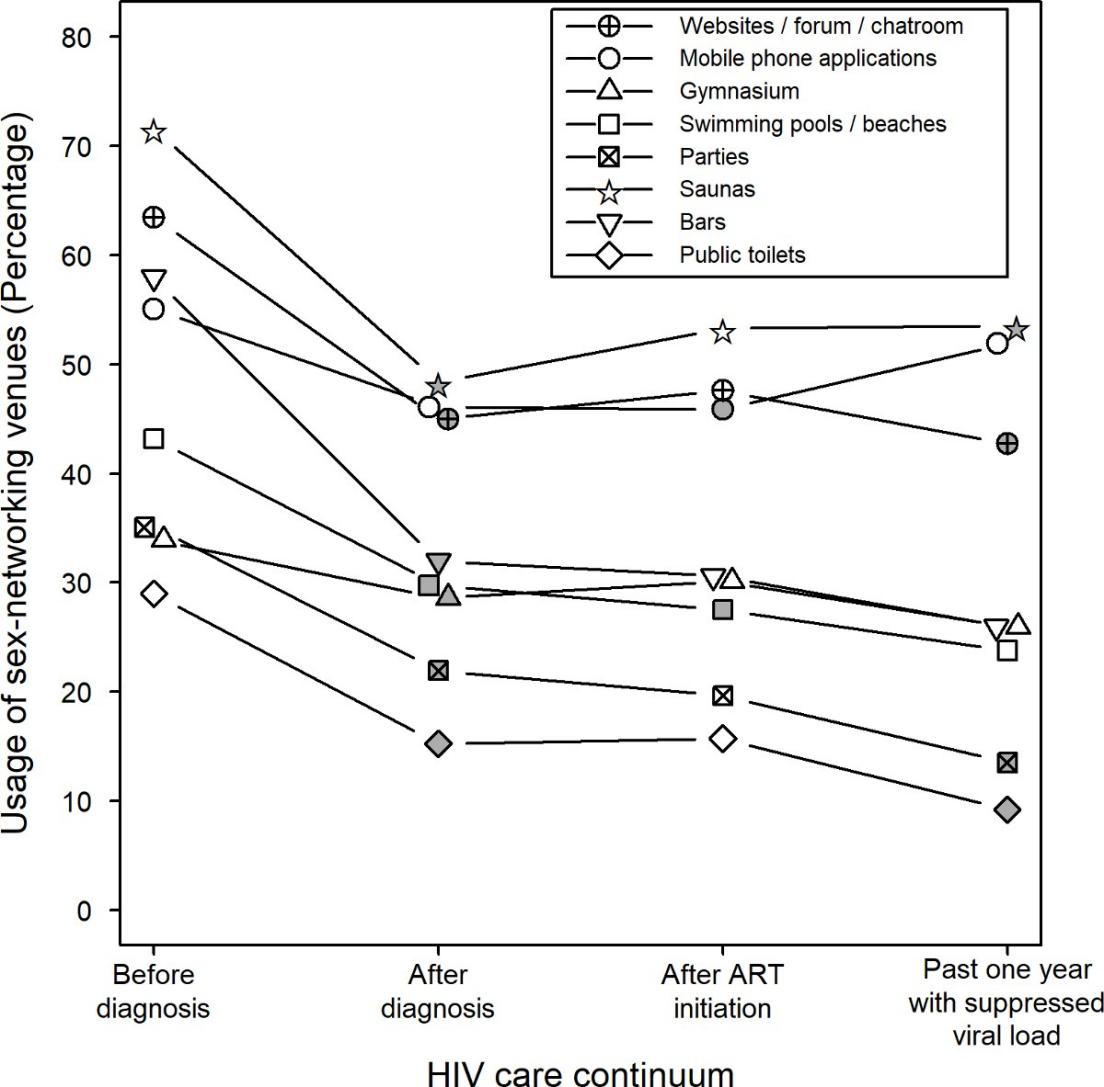
Usage of sex-networking venues at different stages of HIV care continuum. Shaded symbols indicate significant change of usage of sex-networking venue in a modified McNemar’s test, as compared with that in the previous stage of HIV care continuum.

To compare the sexual behaviours before and after HIV diagnosis, a total of 269 respondents, who were diagnosed in or before 2013, were included. Some 76 HIV-positive MSM diagnosed in 2014 were excluded because they did not have a year of behaviour for comparison. Sexual behaviours changed significantly right after HIV diagnosis, in terms of the number of sex partners, condom use, sexual connections and use of alcohol or recreational drug before sex (Table 2 and Fig 1). In the first year after HIV diagnosis, the proportion of MSM having main and casual sex partners fell from 64.9% to 52.8% and from 73.0% to 45.7% respectively. The proportion of MSM with concurrent sex partnership also halved after HIV diagnosis. Inconsistent condom usage rate with either main or casual sex partners dropped significantly from nearly 50% before HIV diagnosis to less than 20% after diagnosis. There was also a significant reduction of alcohol and recreational drug use before anal sex from 53.6% and 36.8% respectively to approximately 25% in the first year after diagnosis. In addition, there was a statistically significant reduction in the usage of seven, out of eight, identified types of sex-networking venues after HIV diagnosis. The exception went to mobile phone applications, which were used by 55.1% of the respondents before HIV diagnosis and 46.1% of MSM after diagnosis (χ^2^ < 0.1, p = 0.91).

**Table 2:** Sexual behaviours at different stages of HIV care continuum.

Sexual characteristics	In the 1-year period before HIV diagnosis (n = 345)		In the 1-year period after HIV diagnosis (n = 269)		Χ^2^	In the 1-year period after ART initiation (n = 229)		Χ^2^	In the past one year with viral load suppressed (n = 185)		Χ^2^
	n	(%)	n	(%)		n	(%)		n	(%)	
**Number of main sex partners**											
0^†^	121	(35.1)	127	(47.2)	5.66*	92	(40.2)	4.43*	69	(37.3)	1.94
1	89	(25.8)	89	(33.1)		80	(34.9)		70	(37.8)	
> 1	135	(39.1)	53	(19.7)		57	(24.9)		46	(24.9)	
**Number of casual sex partners in a month**											
0^†^	93	(27.0)	146	(54.3)	7.13**	112	(48.9)	5.01*	90	(48.6)	0.27
1–2	131	(38.0)	77	(28.6)		83	(36.2)		64	(34.6)	
> 2	121	(35.1)	46	(17.1)		34	(14.8)		31	(16.8)	
**Inconsistent condom use for anal sex with main sex partners**	162	(47.0)	47	(17.5)	6.20*	47	(20.5)	2.38	36	(19.5)	0.28
**Inconsistent condom use for anal sex with casual sex partners**	171	(49.6)	48	(17.8)	7.25**	42	(18.3)	2.30	39	(21.1)	0.41
**Sero-sorting**											
Inclined to seek HIV+ sex partners^†^	-	-	46	(17.1)		39	(17.0)	1.09	25	(13.5)	0
Inclined to seek HIV- sex partners	-	-	23	(8.6)		22	(9.6)		24	(13.0)	
No preference for sex partners of any HIV status	-	-	155	(57.6)		135	(59.0)		113	(61.1)	
Did not have sex	-	-	45	(16.7)		33	(14.4)		23	(12.4)	
**Venues for sex-networking**											
Public toilets	100	(29.0)	41	(15.2)	6.21*	36	(15.7)	1.09	17	(9.2)	8.86**
Bars	200	(58.0)	86	(32.0)	8.01**	70	(30.6)	0.11	48	(25.9)	2.26
Saunas	247	(71.6)	130	(48.3)	7.19**	122	(53.3)	3.38	99	(53.5)	4.41*
Parties	121	(35.1)	59	(21.9)	4.67*	45	(19.7)	2.79	25	(13.5)	7.94**
Swimming pools / beaches	149	(43.2)	80	(29.7)	5.87*	63	(27.5)	4.76*	44	(23.8)	2.48
Gymnasium	117	(33.9)	77	(28.6)	5.03*	69	(30.1)	1.91	48	(25.9)	3.15
Mobile phone applications	190	(55.1)	124	(46.1)	0.01	105	(45.9)	4.17*	96	(51.9)	2.12
Social networking websites / forum / chatroom	219	(63.5)	121	(45.0)	5.05*	109	(47.6)	0.86	79	(42.7)	6.57*
**Concurrent sexual relationship**	204	(59.1)	83	(30.9)	7.50**	72	(31.4)	1.17	50	(27.0)	2.76
**Have ever taken alcohol before anal sex with male**	185	(53.6)	67	(24.9)	7.79**	61	(26.6)	0.33	40	(21.6)	1.30
**Have ever taken recreational drug before anal sex with male**	127	(36.8)	68	(25.3)	7.04**	52	(22.7)	0.90	35	(18.9)	0.11

* *P* value < 0.05

** *P* value < 0.01

^†^ Use as the reference group in Yang’s Chi-square test

The comparison of sexual behaviours before and after initiation of ART was made among 229 MSM, who started their treatment in or before 2013. In other words, 116 MSM, who either started ART after 2013 or had not started ART, were excluded because they did not have a year of behaviour for comparison. Following this transition of the HIV care continuum, the only significant changes in sexual behaviour were the number of main sex partners (χ^2^ = 4.43, p < 0.05) and casual sex partners (χ^2^ = 5.01, p < 0.05). The proportion of MSM having more than two casual sex partners in a month decreased from 17.1% to 14.8%, whereas there was an increase in proportion of MSM having one or two casual sex partners (from 28.6% to 36.2%) in a month during the 1-year period after initiation of ART. However, there was no significant change in condom usage rates, concurrent sexual relationship, usage of six, out of eight, identified types of sex-networking venues and rate of alcohol or recreational drug use before anal sex with male (Table 2 and Fig 1). The proportion of MSM who were inclined to seek HIV-positive sex partners remained at the same level before and after the initiation of ART at about 17%. Around 60% of HIV-positive MSM were not concerned about the HIV status of sex partners.

A total of 185 MSM achieved the status of suppressed viral load in or before 2013. As compared to the time at ART initiation, the major change that occurred after viral load suppression was their usage of sex-networking venues. Following viral suppression, there were a higher number of MSM networking through mobile phone applications, while the usage of public toilets, parties and website for sex-networking had all significantly decreased (Fig 1). No significant difference was found for condom usage rates, number of main or casual sex partners, concurrent sexual partnership and sero-sorting preference after achieving viral load suppression (Table 2).

## Discussion

In this retrospective cross-sectional study covering multiple time-points along the HIV care continuum, substantial changes could be seen in a number of sexual risk behaviours and sexual connections in MSM in Hong Kong following HIV diagnosis, and there was persistently low level of risk behaviours among those in care. These post-infection behaviour profiles, in conjunction with the achievement of viral load suppression among the majority of HIV-positive MSM, likely confer a general reduction of secondary HIV-transmission risk in Hong Kong, which probably does not support the occurrence of extensive HIV transmission.

Our study results echoed the findings reported previously, which showed a significant decline in sexual risk behaviours after HIV diagnosis [10–20]. To describe the transmission patterns and inform public health interventions, sexual risk behaviours among HIV-positive MSM remained one of the top issues in HIV epidemiological studies around the world [31–33]. Yet, such behavioural profiling against the framework of the HIV care continuum has not been reported before. Our study is the first in characterising sexual risk behaviours, as well as sexual connections, among MSM in each stage of the treatment cascade. Despite little changes in sexual risk behaviours after the first year of HIV diagnosis, the rebound of the number of casual sex partners after initiation of ART among our study participants is particularly worrisome in public health context. As reported in previous studies, abstinence was not uncommon among persons newly diagnosed with HIV infection [34–35]. Extrapolating sexual behaviours shortly after HIV diagnosis to those in the ensuing years might not necessarily be appropriate. The observed longitudinal behavioural change in this study, in response to the treatment status, implies the epidemiological importance of long-term monitoring of sexual risk behaviours among HIV-positive MSM, especially when their viral load is not yet suppressed. This implication of the study would be critical in the era of “Undetectable = Untransmittable”, which has been exemplified from the results from The PARTNERS2 Study reporting zero linked HIV transmission from 77,000 condomless-sex acts among 800 serodiscordant couples when the positive partner was on suppressive ART [36]. Knowingly, TasP to reduce HIV incidence would be most relevant when persons with unsuppressed HIV viral load had transmission risk behaviour [37].

Contrary to the moderate changes in sexual risk behaviours, evolvement of sexual connection patterns along the HIV care continuum was more remarkable as elicited in this study. To infer the networking pattern, we had focused on the usage frequency of different venues for seeking male sex partners [25, 38–42]. Again, the major changes of the sexual connections occurred in the first year after HIV diagnosis, while a significant reduction of usage was observed in most identified types of sex-networking venues. The use of mobile phone application was the exception which could be explained by its increasing popularity among MSM in Hong Kong [42]. In this study, the usage of mobile phone applications was found to be more common for virally suppressed MSM. In the mobile era, it is possible that this reflected the general preference shift of sex-networking channels from physical venues to virtual means, and its relationship with treatment status could just be coincidental. Nevertheless, the ever-changing sexual connections shown in this study and heterogeneity of sexual affiliation network among both HIV-positive and HIV-negative MSM shall be addressed when designing HIV prevention strategies [38–39]. Another perspective of sex-networking worth noting in our study was the relatively low level of serosorting among HIV-positive MSM (<20%) in Hong Kong, as compared with studies conducted elsewhere [17]. With the majority of HIV-positive MSM not concerned about the HIV status of their sex partners, the prevalence of unprotected sex in sero-discordant sexual partnership would give a direct impact on HIV transmission, which warrants further investigation.

The HIV transmission potential could also be evaluated in context of the treatment target of “90-90-90” for ending AIDS epidemic, launched by the Joint United Nations Programme on HIV and AIDS [43]. The results from this study indicated the latter two targets had been largely achieved in Hong Kong, as reflected by the initiation of ART in 88% of the diagnosed MSM and viral load suppression among 89% of those on treatment. Taking note of the continued low prevalence of unprotected anal sex after HIV diagnosis, high treatment coverage and achievement of suppressed viral load, our study suggests that delayed or non-diagnosis of HIV infection probably plays a key role in sustaining ongoing virus transmission among MSM in Hong Kong. This finding is in line with the results of a systematic analysis of national HIV treatment cascades, which identified HIV diagnosis as the main gap of existing global HIV care programme [44]. Other modelling studies in the United Kingdom and the United States also suggested that infected but undiagnosed MSM contributed to the majority of new HIV infections, highlighting the vital step for preventing onward transmission by improving HIV testing [21,45]. In the context of the growing HIV incident infections among MSM and low community viral load among diagnosed MSM in Hong Kong, the room for maximising the effectiveness of TasP could exist if HIV testing uptake can be improved. Combined effort on expanding HIV testing for the undiagnosed and maintaining low viral load level for the diagnosed shall be emphasised.

Notwithstanding the fact that most diagnosed HIV-positive MSM had their viral load suppressed, inconsistent condom use among some 20% MSM in this study could predispose to the spread of STI other than HIV infection. This is a phenomenon supported by the recent raised activity or outbreaks of sexually acquired diseases, such as syphilis and viral hepatitis, among HIV-positive MSM [46–48]. As acquisition of STI is normally an indicator of unprotected sex, the epidemiology of co-morbidity with STI among HIV-positive MSM would likely be influenced by the effect of risk compensation of TasP strategy. Irrespective of the level of individual or community viral load, safer sex intervention for STI prevention should never be neglected while implementing TasP for HIV prevention.

This study carries a few limitations. First, recall bias and temporal ambiguity bias on past sexual behaviours of the respondents could not be ruled out in this retrospective cross-sectional study. Though these biases could be resolved by conducting a prospective cohort study, the current study’s design had been adopted in consideration of practicability and ethical feasibility. Second, selection bias might have occurred if patients actively involved in sexual risk behaviours were concerned about discrimination and had refused to join the study. For patients who had joined the study, the possibility of having socially desirable answers from them could not be entirely eliminated. It is likely that such selection bias and information bias have already been minimised by the use of tablet computers in confidence and the emphasis on the anonymity of the questionnaire survey. Third, the interval between HIV diagnosis and initiation of ART among most HIV-positive MSM might not be long enough for behaviour changes to be distinctly recalled. In general, about half of HIV-diagnosed MSM would have been on treatment within a year in Hong Kong. Fourth, it was possible that the differences in behaviours found in this study were influenced by the reducing statistical power caused by the decreasing sample size across different stages of the HIV care continuum. The results about the post-treatment behaviours could also possibly be affected by the smaller sample size, as untreated HIV-positive MSM, who may compensate their risk of transmitting HIV by reducing sexual risk, were excluded. Finally, though the data for this study were obtained from the largest HIV clinic in Hong Kong, these might not be generalisable to the entire MSM community or other populations.

In conclusion, increased condom use and decreased sex-networking were observed among MSM following HIV diagnosis. Sexual risk behaviours and sexual connections changed significantly in the first year after HIV diagnosis, and remained at a similar level afterwards. To prevent secondary HIV transmission in the near future, the importance of early HIV diagnosis and viral load suppression of HIV-positive individuals shall be underscored. The prevalent use of mobile phone applications for sex-networking is a reminder that evolving sexual connection patterns should be taken into consideration while planning public health interventions on controlling HIV spread.

## Supporting information

S1 Text Questionnaire used in the study in both Chinese and English languages. Survey questions included in the study.
(DOCX)
