# Supplementary material for: Changes of sexual risk behaviors and sexual connections among HIV-positive men who have sex with men along their HIV care continuum
Source: PLoS One. 2018 Dec 12;13(12):e0209008. doi: 10.1371/journal.pone.0209008 (PMC6291138; doi:10.1371/journal.pone.0209008)
Supplement: S1 Text — Survey questions included in the study. (DOCX) [file pone.0209008.s001.docx]

**Supporting Information**

**Questionnaire used in the study in both Chinese and English languages**

**甲部‧關於你自己**  病人編號 ____________

1. 你現年 _____ 歲。

2. 你的學歷程度是？ □ 小學 □ 中學 □ 大專、大學或以上

3. 你現時的就業狀況？ □ 全職工作 □ 兼職工作 / 只做Freelance □ 學生
□ 失業 / 失學 □ 退休 □ 其他：_____________

4. 你何時被**診斷**感染愛滋病病毒？ _______________年

5. 確診感染愛滋病病毒前，你接受愛滋病病毒抗體測試的頻率是……
□ 少於3個月一次 □ 約3 – 6個月一次 □ 約6 – 12個月一次
□ 1年以上才一次 □ 不定期接受測試 □ 從未接受過愛滋病病毒抗體測試

6. 你何時首次接受抗病毒藥物治療的時間？
____________年 / □ 尚未開始抗病毒藥物治療

**乙部．關於你確診受感染前一年的生活模式**

1. 你在香港透過以下渠道結識男性性伴侶的頻率是… ( 請於適當方格內打✓ )

| **a. 場地 / 場所** | **從來不去** | **偶然一次/ 超過 3個月一次** | **2 - 3個月 一次** | **1個月一次** | **2 - 3星期 一次** | **1星期 一次** | **1星期 2 - 3次** | **1星期 四次或以上** |
| --- | --- | --- | --- | --- | --- | --- | --- | --- |
| 魚塘 / 公廁 | □ | □ | □ | □ | □ | □ | □ | □ |
| 酒吧 | □ | □ | □ | □ | □ | □ | □ | □ |
| 桑拿 | □ | □ | □ | □ | □ | □ | □ | □ |
| 海灘 / 游泳池 | □ | □ | □ | □ | □ | □ | □ | □ |
| 健身中心 | □ | □ | □ | □ | □ | □ | □ | □ |
| 派對 | □ | □ | □ | □ | □ | □ | □ | □ |
| **b. 互聯網** | **從來不用** | **偶然一次/ 超過 3個月一次** | **2 - 3個月 一次** | **1個月一次** | **2 - 3星期 一次** | **1星期 一次** | **2 - 3日 一次** | **每日都用** |
| 手機應用程式 | □ | □ | □ | □ | □ | □ | □ | □ |
| 社交網站、論壇、 聊天室 | □ | □ | □ | □ | □ | □ | □ | □ |

2. 你確診受感染前一年有沒有情人或固定性伴侶 (如 波友)？
□有 □沒有 (請跳至第3題)

2a. 你確診受感染前一年共有多少個**情人**或**固定性伴侶** (如 **波友**)？ _____ 個

2b. 你與**情人**或**固定性伴侶**發生性關係時，你的安全套使用情況是…

**從來不用 少過一半情況使用 多過一半情況使用 每次都用 不適用**

進行口交時 □ □ □ 　□ 　□

進行肛交時 □ □ □ 　□ 　□

2c.你當年**１個月**內約與**情人**或**固定性伴侶**進行多少次性行為 (口交 / 肛交)？ _____ 次

3. 你確診受感染前一年有沒有非固定性伴侶(不涉及金錢交易，如 一夜情)？
□有 □沒有 (請跳至第4題)

3a. 你當年**１個月**內約共有多少個非固定性伴侶 (如 **一夜情**)？ _____ 個

3b. 你與**非固定性伴侶**發生性關係時，你的安全套使用情況是…

**從來不用 少過一半情況使用 多過一半情況使用 每次都用 不適用**

進行口交時 □ □ □ 　□ 　□

進行肛交時 □ □ □ 　□ 　□

4. 你確診受感染前一年曾否……

|  | **從來 都沒有** | **有 (但極少)** | **間中有** | **經常有** |
| --- | --- | --- | --- | --- |
| a. 於**同一時間與多於一名男性**建立固定的性關係 | □ | □ | □ | □ |
| b. 與男性肛交前曾服用**酒精飲品** | □ | □ | □ | □ |
| c. 與男性肛交前曾服用**軟性藥物** | □ | □ | □ | □ |

**丙部．關於你確診受感染後一年內的生活模式**

1. 與確診受感染前相比，確診受感染後一年內你在香港透過以下渠道結識男性性伴侶的頻率有否改變?

| **a. 場地 / 場所** | **完全 沒有去** | **偶然一次/ 超過 3個月一次** | **2 - 3個月 一次** | **1個月一次** | **2 - 3星期 一次** | **1星期 一次** | **1星期 2 - 3次** | **1星期 四次或以上** |
| --- | --- | --- | --- | --- | --- | --- | --- | --- |
| 魚塘 / 公廁 | □ | □ | □ | □ | □ | □ | □ | □ |
| 酒吧 | □ | □ | □ | □ | □ | □ | □ | □ |
| 桑拿 | □ | □ | □ | □ | □ | □ | □ | □ |
| 海灘 / 游泳池 | □ | □ | □ | □ | □ | □ | □ | □ |
| 健身中心 | □ | □ | □ | □ | □ | □ | □ | □ |
| 派對 | □ | □ | □ | □ | □ | □ | □ | □ |
| **b. 互聯網** | **完全不用** | **偶然一次/ 超過 3個月一次** | **2 - 3個月 一次** | **1個月一次** | **2 - 3星期 一次** | **1星期 一次** | **2 - 3日 一次** | **每日都用** |
| 手機應用程式 | □ | □ | □ | □ | □ | □ | □ | □ |
| 社交網站、論壇、 聊天室 | □ | □ | □ | □ | □ | □ | □ | □ |

2. 你確診受感染後一年內，若仍有性伴侶，各類性伴侶的數目是多少？

情人或固定性伴侶: __________ 個 非固定性伴侶(以一個月計): __________ 個

3. 你確診受感染後一年內，１個月內約與情人或固定性伴侶進行多少次性行為 (口交 / 肛交) ？___________ 次 / □ 沒有情人或固定性伴侶

4. 確診受感染後一年內，你與各類性伴侶發生性關係時，你的安全套使用情況是……

| **與各性伴侶的性行為** | **完全不用** | **少過一半情況使用** | **多過一半情況使用** | **每次都用** | **不適用** |
| --- | --- | --- | --- | --- | --- |
| 與**情人或固定性伴侶**口交 | □ | □ | □ | □ | □ |
| 與**情人或固定性伴侶**肛交 | □ | □ | □ | □ | □ |
| 與　**非固定性伴侶**　口交 | □ | □ | □ | □ | □ |
| 與　**非固定性伴侶**　肛交 | □ | □ | □ | □ | □ |

5. 確診受感染後一年內，你傾向選擇與哪種性伴侶發生性關係？

□ 一定找非感染的性伴侶 (HIV-ve) □ 傾向找非感染的性伴侶 (HIV-ve)

□ 傾向找已感染的性伴侶 (HIV+ve) □ 一定找已感染的性伴侶 (HIV+ve)

□ 沒有理會伴侶是否已感染 □ 不再發生性關係

6. 確診受感染後一年內，你曾否……

|  | **完全沒有** | **有(但極少)** | **間中有** | **經常有** |
| --- | --- | --- | --- | --- |
| a. 於**同一時間與多於一名男性**建立固定的性關係 | □ | □ | □ | □ |
| b. 與男性肛交前曾服用**酒精飲品** | □ | □ | □ | □ |
| c. 與男性肛交前曾服用**軟性藥物** | □ | □ | □ | □ |

**丁部．關於你開始抗病毒藥物治療後 一年內 的生活模式**

**如你1) 於2013或2014年確診感染病毒 或 開始抗病毒藥物治療; 或 2) 確診感染病毒時間與開始抗病毒藥物治療時間在同一年; 或 3) 未開始抗病毒藥物治療，皆可跳過此部分，並請繼續回答戊部問卷。**

1. 與開始抗病毒藥物治療前相比，你在香港透過以下渠道結識男性性伴侶的頻率有否改變？

| **a. 場地 / 場所** | **之前從來不去 治療後也沒有去** | **治療後 沒有再去** | **減少 到訪頻率** | **沒有改變 到訪頻率** | **增加 到訪頻率** | **之前從來不去 但治療後開始去** |
| --- | --- | --- | --- | --- | --- | --- |
| 魚塘 / 公廁 | □ | □ | □ | □ | □ | □ |
| 酒吧 | □ | □ | □ | □ | □ | □ |
| 桑拿 | □ | □ | □ | □ | □ | □ |
| 海灘 / 游泳池 | □ | □ | □ | □ | □ | □ |
| 健身中心 | □ | □ | □ | □ | □ | □ |
| 派對 | □ | □ | □ | □ | □ | □ |
| **b. 互聯網** | **之前從來不用 治療後也沒有用** | **治療後 沒有再用** | **減少 使用頻率** | **沒有改變 使用頻率** | **增加 使用頻率** | **之前從來不用 但治療後開始用** |
| 手機應用程式 | □ | □ | □ | □ | □ | □ |
| 社交網站、論壇、聊天室 | □ | □ | □ | □ | □ | □ |

2a. 與開始抗病毒藥物治療前相比，你各類性伴侶的數目轉變情況是…… ( 請於最適當的方格內打✓ )

| **性伴侶** | **之前沒有 治療後也沒有** | **之前有 治療後再沒有** | **治療後 數目減少** | **治療後數目 沒有大改變** | **治療後 數目增加** | **之前沒有 但治療後開始有** |
| --- | --- | --- | --- | --- | --- | --- |
| 情人 或 固定性伴侶 | □ | □ | □ | □ | □ | □ |
| 非固定性伴侶 | □ | □ | □ | □ | □ | □ |

2b. 開始抗病毒藥物治療後一年內，若仍有性伴侶，各類性伴侶的數目是多少？

情人或固定性伴侶: _________ 個 非固定性伴侶(以一個月計): ________ 個

2c. 開始抗病毒藥物治療後一年內，你平均1個月內與情人或固定性伴侶進行多少次性行為 (口交 / 肛交)? __________次 / □ 沒有情人或固定性伴侶

3. 開始抗病毒藥物治療後一年內，你與各類性伴侶發生性關係時，你的安全套使用情況是……

| **與各性伴侶的性行為** | **治療後 從來 不用** | **少於一半 情況使用 並明顯 比治療前少** | **少於一半 情況使用 但明顯 比治療前多** | **治療前後使用情況相若** | **多於一半 情況使用 但明顯 比治療前少** | **多於一半 情況使用 並明顯 比治療前多** | **治療後 每次 都用** | **不適用** |
| --- | --- | --- | --- | --- | --- | --- | --- | --- |
| 與**情人或固定性伴侶**口交 | □ | □ | □ | □ | □ | □ | □ | □ |
| 與**情人或固定性伴侶**肛交 | □ | □ | □ | □ | □ | □ | □ | □ |
| 與**非固定性伴侶**口交 | □ | □ | □ | □ | □ | □ | □ | □ |
| 與**非固定性伴侶**肛交 | □ | □ | □ | □ | □ | □ | □ | □ |

4. 開始抗病毒藥物治療後一年內，你傾向選擇與哪種性伴侶發生性關係？

□ 一定找非感染的性伴侶 (HIV-ve) □ 傾向找非感染的性伴侶 (HIV-ve)

□ 傾向找已感染的性伴侶 (HIV+ve) □ 一定找已感染的性伴侶 (HIV+ve)

□ 沒有理會伴侶是否已感染 □ 不再發生性關係

5. 開始抗病毒藥物治療後一年內，你曾否……

|  | **完全沒有** | **有 (比治療前少)** | **有 (情況與治療前相若)** | **有 (比治療前多)** |
| --- | --- | --- | --- | --- |
| a. 於**同一時間與多於一名男性**建立固定的性關係 | □ | □ | □ | □ |
| b. 與男性肛交前曾服用**酒精飲品** | □ | □ | □ | □ |
| c. 與男性肛交前曾服用**軟性藥物** | □ | □ | □ | □ |

**戊部．關於你過去一年內的生活模式**

**如你於2013年或2014年確診感染愛滋病病毒，可跳過此部分。**

1. 你在香港透過以下渠道結識男性性伴侶的頻率是… ( 請於適當方格內打✓ )

| **a. 場地 / 場所** | **完全 沒有去** | **偶然一次/ 超過 3個月一次** | **2 - 3個月 一次** | **1個月一次** | **2 - 3星期 一次** | **1星期 一次** | **1星期 2 - 3次** | **1星期 四次或以上** |
| --- | --- | --- | --- | --- | --- | --- | --- | --- |
| 魚塘 / 公廁 | □ | □ | □ | □ | □ | □ | □ | □ |
| 酒吧 | □ | □ | □ | □ | □ | □ | □ | □ |
| 桑拿 | □ | □ | □ | □ | □ | □ | □ | □ |
| 海灘 / 游泳池 | □ | □ | □ | □ | □ | □ | □ | □ |
| 健身中心 | □ | □ | □ | □ | □ | □ | □ | □ |
| 派對 | □ | □ | □ | □ | □ | □ | □ | □ |
| **b. 互聯網** | **完全不用** | **偶然一次/ 超過 3個月一次** | **2 - 3個月 一次** | **1個月一次** | **2 - 3星期 一次** | **1星期 一次** | **2 - 3日 一次** | **每日都用** |
| 手機應用程式 | □ | □ | □ | □ | □ | □ | □ | □ |
| 社交網站、論壇、 聊天室 | □ | □ | □ | □ | □ | □ | □ | □ |

2. 過去一年，你有沒有情人或固定性伴侶 (如 波友)？□有 □沒有 (請跳至第3題)

2a. 過去一年，你共有多少個**情人**或**固定性伴侶**(如 **波友**)？ ____________ 個

2b. 過去一年，你與**情人**或**固定性伴侶**發生性關係時，你的安全套使用情況是…

**完全不用 少過一半情況使用 多過一半情況使用 每次都用 不適用**

進行口交時 □ □ □ 　□ 　□

進行肛交時 □ □ □ 　□ 　□

2c. 過去一年，你**１個月**內約與**情人**或**固定性伴侶**進行多少次性行為 (口交 / 肛交)？_______ 次

3. 過去一年，你有沒有非固定性伴侶(不涉及金錢交易，如 一夜情)？

□有 □沒有 (請跳至第4題)

3a. 過去一年，你**１個月**內約共有多少個非固定性伴侶 (如 **一夜情**)？ __________ 個

3b. 過去一年，你與**非固定性伴侶**發生性關係時，你的安全套使用情況是…

**完全不用 少過一半情況使用 多過一半情況使用 每次都用 不適用**

進行口交時 □ □ □ 　□ 　□

進行肛交時 □ □ □ 　□ 　□

4. 過去一年，你傾向選擇與哪種性伴侶發生性關係？

□ 一定找非感染的性伴侶 (HIV-ve) □ 傾向找非感染的性伴侶 (HIV-ve)

□ 傾向找已感染的性伴侶 (HIV+ve) □ 一定找已感染的性伴侶 (HIV+ve)

□ 沒有理會伴侶是否已感染 □ 不再發生性關係

5.. 過去一年，你曾否

|  | **完全沒有** | **有(但極少)** | **間中有** | **經常有** |
| --- | --- | --- | --- | --- |
| a. 於**同一時間與多於一名男性**建立固定的性關係 | □ | □ | □ | □ |
| b. 與男性肛交前曾服用**酒精飲品** | □ | □ | □ | □ |
| c. 與男性肛交前曾服用**軟性藥物** | □ | □ | □ | □ |

**Section A Demographic information** Patient no. ____________

1. How old are you? _____ years old

2. Highest education level attained:
□ Primary □ Secondary □ Post-secondary / University or above

3. What is your current socio-economic status?
□ Full-time employed / self-employed □ Part-time employed / Freelancer
□ Student □ Unemployed
□ Retired □ Others: _______________

4. When were you **diagnosed** as HIV-positive? ________ (yyyy)

5. How often did you test for HIV before diagnosis of HIV infection?
□ Once less than 3 months □ Every 3 – 6 months
□ Every 6 – 12 months □ Once more than 1 year
□ Non-regular testing □ I had never tested for HIV

6. When was your **antiretroviral therapy initiated**?
________________ (yyyy) / □ I haven’t started antiretroviral therapy yet

**Section B Lifestyle in the 1-year period before HIV diagnosis**

1. How often did you seek male sex partner(s) in Hong Kong through the following channels?

| **a. Social venues / functions** | **Never** | **Occasionally/ less than once every 3 months** | **Every 2-3 months** | **Every month** | **Every 2-3 weeks** | **Every week** | **2-3 times per week** | **4 times or more per week** |
| --- | --- | --- | --- | --- | --- | --- | --- | --- |
| Public toilets | □ | □ | □ | □ | □ | □ | □ | □ |
| Bar | □ | □ | □ | □ | □ | □ | □ | □ |
| Sauna | □ | □ | □ | □ | □ | □ | □ | □ |
| Beach / Swimming pool | □ | □ | □ | □ | □ | □ | □ | □ |
| Gymnasium | □ | □ | □ | □ | □ | □ | □ | □ |
| Party | □ | □ | □ | □ | □ | □ | □ | □ |
| **b. Internet** | **Never** | **Occasionally/ less than once every 3 months** | **Every 2-3 months** | **Every month** | **Every 2-3 weeks** | **Every week** | **Once per 2-3 days** | **Every day** |
| Mobile phone applications | □ | □ | □ | □ | □ | □ | □ | □ |
| Social networking websites, forum or chat-room | □ | □ | □ | □ | □ | □ | □ | □ |

2. In the 1-year period before HIV diagnosis, did you have any lover(s) or regular sex partner(s)? □ Yes □ No [skip to Question 3]

2a. How many **lover(s)** or **regular male sex partner(s)** did you have in that year? [ ______ ]

2b. How often did you use a condom when you were having sex with your **lover(s)** or **regular sex partner(s)**?

**Never Less than half of those occasions More than half of those occasion Always N/A**

Oral sex □ □ □ □ □

Anal sex □ □ □ □ □

2c. How many times did you have sex (oral or anal sex) with your **lover(s)** or **regular sex partner(s)** in a month? [ ______________ ]

3. In the 1-year period before HIV diagnosis, did you have sex with non-regular partner(s) without involving money or other rewards (e.g. one-night stand)?
□ Yes □ No [skip to Question 4]

3a. How many **non-regular male sex partner(s)** (e.g. one-night stand partner) did you have for each month? [ ______________ ]

3b. How often did you use a condom when you were having sex with your **non-regular sex partner(s)**?

**Never Less than half of those occasions More than half of those occasion Always N/A**

Oral sex □ □ □ □ □

Anal sex □ □ □ □ □

4. In the 1-year period before HIV diagnosis, had you ever …

|  | **No** | **Yes, but rarely** | **Yes, sometimes** | **Yes, I always did so** |
| --- | --- | --- | --- | --- |
| a. established regular sexual relationship with **more than one man simultaneously** | □ | □ | □ | □ |
| b. taken any **alcoholic drinks** before having anal sex with male | □ | □ | □ | □ |
| c. taken any **recreational drugs** before having anal sex with male | □ | □ | □ | □ |

**Section C Lifestyle in the 1-year period after HIV diagnosis**

1. In the 1-year period after HIV diagnosis, did you change the use of the following venues for seeking male sex partner(s) in Hong Kong, as compared with that before diagnosis?

| **a. Social venues / functions** | **Never** | **Occasionally / less than once every 3 months** | **Every 2 – 3 months** | **Every month** | **Every 2 – 3 weeks** | **Every week** | **2 – 3 times per week** | **4 times or more per week** |
| --- | --- | --- | --- | --- | --- | --- | --- | --- |
| Public toilets | □ | □ | □ | □ | □ | □ | □ | □ |
| Bar | □ | □ | □ | □ | □ | □ | □ | □ |
| Sauna | □ | □ | □ | □ | □ | □ | □ | □ |
| Beach / Swimming pool | □ | □ | □ | □ | □ | □ | □ | □ |
| Gymnasium | □ | □ | □ | □ | □ | □ | □ | □ |
| Party | □ | □ | □ | □ | □ | □ | □ | □ |
| **b. Internet** | **Never** | **Occasionally / less than once every 3 months** | **Every 2 – 3 months** | **Every month** | **Every 2 – 3 weeks** | **Every week** | **2 – 3 times per week** | **4 times or more per week** |
| Mobile phone applications | □ | □ | □ | □ | □ | □ | □ | □ |
| Social networking websites, forum or chat-room | □ | □ | □ | □ | □ | □ | □ | □ |

2. In the 1-year period after HIV diagnosis,
if you still had male sex partner(s) after HIV diagnosis, how many did you have?

Lover(s) / regular sex partner(s): [ _________ ]

Non-regular sex partner(s) (for each month): [ ________ ]

3. In the 1-year period after HIV diagnosis, how many times did you have sex (oral or anal sex) with your lover(s) or regular sex partner(s) in a month?
[_____________] / □ I didn’t have any lover(s) or regular sex partner(s).

4. In the 1-year period after HIV diagnosis,
how often did you use a condom when having sex with different types of sex partners?

| **Sex acts with  different types of sex partners** | **Never** | **Less than half of those occasions** | **Half or more of those occasions** | **Every time** | **N/A** |
| --- | --- | --- | --- | --- | --- |
| Oral sex with **lovers / regular partners** | □ | □ | □ | □ | □ |
| Anal sex with **lovers / regular partners** | □ | □ | □ | □ | □ |
| Oral sex with **non-regular partners** | □ | □ | □ | □ | □ |
| Anal sex with **non-regular partners** | □ | □ | □ | □ | □ |

5. In the 1-year period after HIV diagnosis, which type of sex partners were you inclined to have sex with?

□ I definitely looked for a HIV-ve sex partner.

□ I was inclined to find a HIV-ve sex partner.

□ I was inclined to find a HIV+ve sex partner.

□ I definitely looked for a HIV+ve sex partner.

□ I did not care the HIV status of my sex partner(s).

□ I did not have sex any more.

6. In the 1-year period after HIV diagnosis, had you ever …

|  | **No** | **Yes,  but rarely** | **Yes, sometimes** | **Yes, I always did so** |
| --- | --- | --- | --- | --- |
| a. established regular sexual relationship with **more than one man simultaneously** | □ | □ | □ | □ |
| b. taken any **alcoholic drinks** before having anal sex with male | □ | □ | □ | □ |
| c. taken any **recreational drugs** before having anal sex with male | □ | □ | □ | □ |

**Section D Lifestyle in the 1-year period after initiation of antiretroviral therapy**

**If you 1) were diagnosed or have initiated antiretroviral therapy (ART) in 2013 or 2014, 2) have initiated ART in the same year as HIV diagnosis, or 3) have not started ART, please skip this section and continue with Section E.**

1. Did you have any change in frequency of seeking male sex partner(s) in Hong Kong through the following channels after initiation of antiretroviral therapy, as compared with that before receiving medications?

| **a. Social venues / functions** | **Never visit these places before and after initiation of treatment** | **No longer visit these places after initiation of treatment** | **Decrease in visit frequency** | **No change in visit frequency** | **Increase in visit frequency** | **Never before receiving treatment but start visiting these places after treatment started** |
| --- | --- | --- | --- | --- | --- | --- |
| Public toilets | □ | □ | □ | □ | □ | □ |
| Bar | □ | □ | □ | □ | □ | □ |
| Sauna | □ | □ | □ | □ | □ | □ |
| Beach / Swimming pool | □ | □ | □ | □ | □ | □ |
| Gymnasium | □ | □ | □ | □ | □ | □ |
| Party | □ | □ | □ | □ | □ | □ |
| **b. Internet** | **Never use these channels before and after initiation of treatment** | **No longer use these channels after initiation of treatment** | **Decrease in usage frequency** | **No change in usage frequency** | **Increase in usage frequency** | **Never before receiving treatment but start using these channels after treatment started** |
| Mobile phone applications | □ | □ | □ | □ | □ | □ |
| Social networking websites, forum or chat-room | □ | □ | □ | □ | □ | □ |

2a. Did you have any change in number of male sex partner(s) after initiation of antiretroviral therapy, as compared with that before receiving medications? (Check the most appropriate boxes)

| **Type of sex partners** | **Never have this type of sex partners before and after initiation of treatment** | **No longer having this type of sex partners after initiation of treatment** | **Decrease in number after treatment started** | **No change in number after treatment started** | **Increase in number after treatment started** | **Start having this type of sex partners after initiation of treatment** |
| --- | --- | --- | --- | --- | --- | --- |
| Lover / regular sex partner | □ | □ | □ | □ | □ | □ |
| Non-regular sex partner | □ | □ | □ | □ | □ | □ |

2b. If you still had male sex partner(s) after initiation of antiretroviral therapy, how many did you have?

Lover(s) / regular sex partner(s): [ ________ ]

Non-regular sex partner(s) (for each month): [ ________ ]

2c. In the 1-year period after initiation of antiretroviral therapy, how many times did you have sex (oral or anal sex) with your lover(s) or regular sex partner(s) in a month?
[__________] / □ I didn’t have any lover(s) or regular sex partner(s).

3. After treatment started, how often did you use a condom when having sex with different types of sex partners?

| **Sex acts with**  **different types of sex partners** | **Never** | **Less than half  of those occasions, and** | | **Condom usage rate is similar with that before treatment started** | **More than half  of those occasions, and** | | **Always** | **N/A** |
| --- | --- | --- | --- | --- | --- | --- | --- | --- |
|  |  | **Significantly less often than that before treatment started** | **Significantly more often than that before treatment started** |  | **Significantly less often than that before treatment started** | **Significantly more often than that before treatment started** |  |  |
| Oral sex with **lovers / regular partners** | □ | □ | □ | □ | □ | □ | □ | □ |
| Anal sex with **lovers / regular partners** | □ | □ | □ | □ | □ | □ | □ | □ |
| Oral sex with **non-regular partners** | □ | □ | □ | □ | □ | □ | □ | □ |
| Anal sex with **non-regular partners** | □ | □ | □ | □ | □ | □ | □ | □ |

4. In the 1-year period after initiation of antiretroviral therapy, which type of sex partners were you inclined to have sex with?

□ I definitely looked for a HIV-ve sex partner.

□ I was inclined to find a HIV-ve sex partner.

□ I was inclined to find a HIV+ve sex partner.

□ I definitely looked for a HIV+ve sex partner.

□ I did not care the HIV status of my sex partner(s).

□ I did not have sex any more.

5. After initiation of antiretroviral therapy, had you ever …

|  | **No** | **Yes, but less often than that before initiation of treatment** | **Yes, the frequency is similar with that before initiation of treatment** | **Yes, and more often than that before initiation of treatment** |
| --- | --- | --- | --- | --- |
| a. established regular sexual relationship with **more than one man simultaneously** | □ | □ | □ | □ |
| b. taken any **alcoholic drinks** before having anal sex with male | □ | □ | □ | □ |
| c. taken any **recreational drugs** before having anal sex with male | □ | □ | □ | □ |

**Section E Lifestyle in the past one year**

**If you were diagnosed with HIV in 2013 or 2014, please skip this section.**

1. How often did you seek male sex partner(s) in Hong Kong through the following channels?

| **a. Social venues / functions** | **Never** | **Occasionally/ less than once every 3 months** | **Every 2-3 months** | **Every month** | **Every 2-3 weeks** | **Every week** | **2-3 times per week** | **4 times or more per week** |
| --- | --- | --- | --- | --- | --- | --- | --- | --- |
| Public toilets | □ | □ | □ | □ | □ | □ | □ | □ |
| Bar | □ | □ | □ | □ | □ | □ | □ | □ |
| Sauna | □ | □ | □ | □ | □ | □ | □ | □ |
| Beach / Swimming pool | □ | □ | □ | □ | □ | □ | □ | □ |
| Gymnasium | □ | □ | □ | □ | □ | □ | □ | □ |
| Party | □ | □ | □ | □ | □ | □ | □ | □ |
| **b. Internet** | **Never** | **Occasionally/ less than once every 3 months** | **Every 2-3 months** | **Every month** | **Every 2-3 weeks** | **Every week** | **Once per 2-3 days** | **Every day** |
| Mobile phone applications | □ | □ | □ | □ | □ | □ | □ | □ |
| Social networking websites, forum or chat-room | □ | □ | □ | □ | □ | □ | □ | □ |

2.In the past one year, did you have any lover(s) or regular sex partner(s)?

□ Yes □ No [skip to Question 3]

2a. How many **lover(s)** or **regular male sex partner(s)** did you have in the past one year?

[ _____________ ]

2b. How often did you use a condom when you were having sex with your **lover(s)** or **regular sex partner(s)**?

**Never Less than half of those occasions More than half of those occasion Always N/A**

Oral sex □ □ □ □ □

Anal sex □ □ □ □ □

2c. How many times did you have sex (oral or anal sex) with your **lover(s)** or **regular sex partner(s)** in a month? [ ____________________ ]

3. In the past one year, did you have sex with non-regular partner(s) without involving money or other rewards (e.g. one-night stand)? □ Yes □ No [skip to Question 5]

3a. How many **non-regular male sex partner(s)** (e.g. one-night stand partner) did you have for each month? [ ____________________ ]

3b. How often did you use a condom when you were having sex with your **non-regular sex partner(s)**?

**Never Less than half of those occasions More than half of those occasion Always N/A**

Oral sex □ □ □ □ □

Anal sex □ □ □ □ □

4. In the past one year, which type of sex partners were you inclined to have sex with?

□ I definitely looked for a HIV-ve sex partner.

□ I was inclined to find a HIV-ve sex partner.

□ I was inclined to find a HIV+ve sex partner.

□ I definitely looked for a HIV+ve sex partner.

□ I did not care the HIV status of my sex partner(s).

□ I did not have sex any more

5. In the past one year, have you ever …

|  | **No** | **Yes,  but rarely** | **Yes, sometimes** | **Yes, I always did so** |
| --- | --- | --- | --- | --- |
| a. established regular sexual relationship with **more than one man simultaneously** | □ | □ | □ | □ |
| b. taken any **alcoholic drinks** before having anal sex with male | □ | □ | □ | □ |
| c. taken any **recreational drugs** before having anal sex with male | □ | □ | □ | □ |
